# Stage-Specific Pathways of *Leishmania infantum chagasi* Entry and Phagosome Maturation in Macrophages

**DOI:** 10.1371/journal.pone.0019000

**Published:** 2011-04-28

**Authors:** Nilda E. Rodríguez, Upasna Gaur Dixit, Lee-Ann H. Allen, Mary E. Wilson

**Affiliations:** 1 Veterans' Affairs Medical Center, Iowa City, Iowa, United States of America; 2 Department of Internal Medicine, University of Iowa, Iowa City, Iowa, United States of America; 3 Department of Microbiology, University of Iowa, Iowa City, Iowa, United States of America; 4 Department of Epidemiology, University of Iowa, Iowa City, Iowa, United States of America; University of Birmingham, United Kingdom

## Abstract

The life stages of *Leishmania* spp. include the infectious promastigote and the replicative intracellular amastigote. Each stage is phagocytosed by macrophages during the parasite life cycle. We previously showed that caveolae, a subset of cholesterol-rich membrane lipid rafts, facilitate uptake and intracellular survival of virulent promastigotes by macrophages, at least in part, by delaying parasitophorous vacuole (PV)-lysosome fusion. We hypothesized that amastigotes and promastigotes would differ in their route of macrophage entry and mechanism of PV maturation. Indeed, transient disruption of macrophage lipid rafts decreased the entry of promastigotes, but not amastigotes, into macrophages (P<0.001). Promastigote-containing PVs were positive for caveolin-1, and co-localized transiently with EEA-1 and Rab5 at 5 minutes. Amastigote-generated PVs lacked caveolin-1 but retained Rab5 and EEA-1 for at least 30 minutes or 2 hours, respectively. Coinciding with their conversion into amastigotes, the number of promastigote PVs positive for LAMP-1 increased from 20% at 1 hour, to 46% by 24 hours, (P<0.001, Chi square). In contrast, more than 80% of amastigote-initiated PVs were LAMP-1+ at both 1 and 24 hours. Furthermore, lipid raft disruption increased LAMP-1 recruitment to promastigote, but not to amastigote-containing compartments. Overall, our data showed that promastigotes enter macrophages through cholesterol-rich domains like caveolae to delay fusion with lysosomes. In contrast, amastigotes enter through a non-caveolae pathway, and their PVs rapidly fuse with late endosomes but prolong their association with early endosome markers. These results suggest a model in which promastigotes and amastigotes use different mechanisms to enter macrophages, modulate the kinetics of phagosome maturation, and facilitate their intracellular survival.

## Introduction

The *Leishmania* spp. are pathogenic protozoa that cause endemic human disease in tropical and subtropical countries. *Leishmania* transform between two distinct life cycle stages, the infective promastigote and the intracellular amastigote. During a blood meal, a sand fly vector inoculates promastigotes into the skin of the mammalian host, whereupon they are taken up by macrophages and convert into amastigotes. Amastigotes replicate intracellularly and spread to new macrophages, disseminating and causing disease [1], [2].

Many studies of *Leishmania* phagocytosis have addressed the interactions between macrophages and promastigotes. The promastigote surface metalloprotease GP63 (also called MSP) facilitates parasite entry through the third complement receptor CR3, which binds iC3b and mediates pathogen uptake without eliciting robust microbial responses [1], [3], [4]. Amastigotes have been shown to enter macrophages after ligating Fc-γ and phosphatidylserine (PS) receptors which induce TGF-β and IL-10 production, resulting in decreased classical macrophage activation and enhanced parasite survival [5], [6].

We previously showed that phagocytosis of *Leishmania* proceeds through a subset of lipid-enriched membrane microdomains called caveolae, which are enriched in cholesterol, ganglioside M-1 (GM-1), GPI anchored proteins and caveolins-1, -2 and -3 [7]–[9]. *Leishmania infantum chagasi* infection increases the abundance of transcripts encoding several proteins of caveolae, including dynamin-2 and caveolins-1 and -3 [10]. Furthermore, the caveolae markers GM-1 and caveolin-1 [7]–[9] cluster around the phagosome during uptake of virulent lines of *L. i. chagasi* and continue to co-localize with the parasites for up to 24 h. Similar to other pathogens, promastigotes in these caveolae decorated compartments delay fusion with lysosomes for 24–48 h after phagocytosis [11]–[14]. However, disruption of macrophage lipid rafts prior to phagocytosis of virulent promastigotes decreases promastigote uptake and intracellular survival, and accelerates the rate of phagosome–lysosome fusion. Thus, disruption of caveolae alters the kinetics of maturation of vacuoles containing virulent promastigotes such that they resemble phagosomes containing attenuated promastigotes [9].

Lipophosphoglycan (LPG) is a promastigote-specific virulence factor that facilitates parasite survival by delaying fusion of the parasitophorous vacuole (PV) with lysosomes and impairing local superoxide production [11]–[15]. Amastigotes lack LPG and as predicted, amastigote-containing phagosomes rapidly acquire lysosomal markers after phagocytosis [3], [4], [11], [16]. A model of *L. amazonensis* infection suggests that phagosomes containing promastigotes and amastigotes acquire lysosomal markers with different kinetics [17]. Contrary to promastigotes, amastigotes survive and replicate in the phagolysosome suggesting that lysosomal fusion does not impair amastigote survival [2], [18].

Differences between the surface molecules displayed and the macrophage receptors targeted by each of the *Leishmania* life stages [19], [20]–[22] led us to hypothesize that promastigotes and amastigotes may differ in their ability to use cholesterol-rich microdomains to enter macrophages. We further hypothesized that there would be corresponding differences in the rates of phagosome maturation and intracellular survival. In support of this hypothesis, we showed that, in contrast to promastigotes, amastigote phagocytosis was not dependent on intact lipid rafts and did not proceed through caveolae. Nonetheless, depletion of cholesterol-rich domains on the macrophage surface impaired the long-term ability of amastigotes to replicate. Further investigations revealed that amastigote-induced PVs retained early endosome markers, even though they rapidly acquired LAMP-1. This suggests that processes other than avoidance of PV-lysosomal fusion contribute to the intracellular survival of leishmania.

## Materials and Methods

### Wild-type parasites

A Brazilian strain of *L. chagasi* (MHOM/BR/00/1669) was maintained by serial passage in male Syrian hamsters and used within 3 weeks of isolation from hamster spleens for experiments [23]. Promastigotes were grown in hemoflagellate-modified minimal essential medium (HOMEM) with 10% HI-FCS until reaching stationary phase after 7–9 days [24], [25]. Metacyclic promastigotes were isolated from stationary cultures according to their density, using a Ficoll-Hypaque (Sigma St. Louis, MO) gradient separation method as described [26]. Amastigotes were isolated from the spleens of infected male Syrian hamsters and incubated for 24 h at 37°C in amastigote growth medium containing 20% FCS at 37°C, 5% CO_2_, pH 5.5 [25], [27]. After 24 h, cultures were suspended in HBSS and centrifuged at 57× g, 10 minutes to remove debris. Amastigotes were pelleted at 2060× g. This method allows spontaneous detachment of host PV membranes and the isolation of pure amastigote cultures [28].

### LcJ parasites

The LcJ parasite line, derived from wild-type *L.i. chagasi*, converts between promastigote and amastigote in axenic culture. LcJ promastigotes and amastigotes were maintained in their respective media, and switched from one stage to the other every three weeks [25]. To ensure that LcJ promastigotes or amastigotes were fully converted, all experiments were performed with parasites passed three times in conditions specific for each stage. Logarithmic and stationary phase promastigote populations were previously defined by cell density and morphology [29].

### Ethics Statement

All procedures involving animals have been performed according to the ethical guidelines of the National Institute of Health and the Chief Veterinary Office of the Department of Veterans' Affairs. Procedures were approved by the Institutional Animal Care and Use Committees (IACUC) of the University of Iowa, the Iowa City VA Medical Center, and the Chief Veterinary Office of the Department of Veterans' Affairs. The approved IACUC animal protocols are # 7091201 for mice and # 7091202 for hamsters.

### Bone marrow macrophages

Bone marrow cells from BALB/c mouse femurs were cultured at 37°C, 5% CO_2_ in RP-10 [10% heat-inactivated fetal calf serum, 2 mM L-glutamine, 100 U/ml penicillin, and 50 µg/ml streptomycin in RPMI-1640 (Gibco, Carlsbad, CA)] containing 20% cell culture supernatant from L929 cells (American Tissue Type Collection, Manassas, VA) as a source of macrophage colony-stimulating factor. After 7–9 days, differentiated adherent macrophages were detached from the plate with 2.5 mg/ml trypsin plus 1 mM EDTA (Gibco) [30]. Glass coverslips in 24 well plates were seeded overnight with 5×10^5^ macrophages and either left untreated or treated with MβCD as described [9]. Briefly, cells were incubated with 10 mM MβCD in the absence of serum for 1 h, then rinsed twice with PBS and incubated in RPMI for parasite infections.

### Macrophage infections

Macrophages were infected with either promastigotes at a multiplicity of infection (MOI) of 10∶1 or amastigotes at a 3∶1 MOI. Unless stated otherwise, experiments were performed using non-opsonized parasites. For some experiments, parasites were opsonized with 5% A/J (C5-deficient) serum. Opsonized promastigotes were used at 5∶1 MOI and amastigotes at 2∶1 MOI. Binding was synchronized by centrifugation (3 min, 330× g, 4°C) and warmed to 37°C, 5% CO_2_ to initiate uptake. Infected macrophages were incubated in 5% CO_2_, 37°C in RP-10. Extracellular parasites were removed by rinsing twice with PBS after 30 min or less for shorter infections, and incubated in fresh RP-10 [9], [10]. At the indicated time points, coverslips were blown dry and stained with Diff Quik (Wright-Giemsa). Intracellular parasites were enumerated by light microscopy.

### Confocal microscopy

Macrophages were seeded at 5×10^5^ on 12 mm coverslips and infected with *L. i. chagasi* [9], [10]. In some experiments parasites were stained with carboxy-fluorescein diacetate succinimidyl ester (CFSE) as described [31]. At appropriate time points, cells were rinsed twice with PBS and fixed in 2% paraformaldehyde (30 min; EMS, Hatfield, PA), permeabilized in 0.2% Triton X-100 (15 min), incubated in 50 mM glycine (15 min), and blocked in 5% non-fat dry milk/PBS (30 min) or 5% normal goat serum. Macrophages were incubated with primary antibodies overnight at 4°C, rinsed, and then incubated with secondary antibodies for 1 h at room temperature. After rinsing in PBS and mounting with Vectashield H-1000 (Vector Labs, Burlingame, CA), slides were examined on a Zeiss 510 laser scanning confocal microscope running version 3.2 software (Carl Zeiss, Inc., Thornwood, NY). Confocal optical sections were analyzed using the LSM 5 image browser. All microscopic studies were performed at the University of Iowa Central Microscopy Research Facility.

Primary antibodies were rabbit anti-EEA-1 (Calbiochem), mouse monoclonal anti-Rab5 (Santa Cruz SC-46692), and rat 1D4B anti-LAMP-1 (developed by J. Thomas August, Developmental Studies Hybridoma Bank, University of Iowa). All primary antibodies were used at 1∶100. Secondary antibodies were Alexa Fluor 568 (red) goat anti-rabbit IgG or Alexa Fluor 647 (blue) goat anti-rabbit (Molecular Probes) were used at 1∶200. DNA was stained with TOPRO-3 (Molecular Probes) at a 1∶1500 dilution. Actin was stained with Alexa Fluor 647 (blue) Phalloidin (Invitrogen) at 1∶50.

### Colocalization

Assessment of colocalization events was done with Image J. In particular, selected pixels with a ratio ≥50% and a Pearson's Rr value ≥0.6 were considered to be colocalized.

### Statistical analyses

Statistical analysis for T-test (light microscopy repeats) or Chi square (pooled confocal micrographs) were performed using the Sigma Stat 3.1 or GraphPad Prism 5 programs, respectively.

## Results

### Intact cholesterol-containing lipid rafts facilitate the entry and intracellular survival of wild-type *L. i. chagasi* promastigotes but not amastigotes

**Figure 1:**
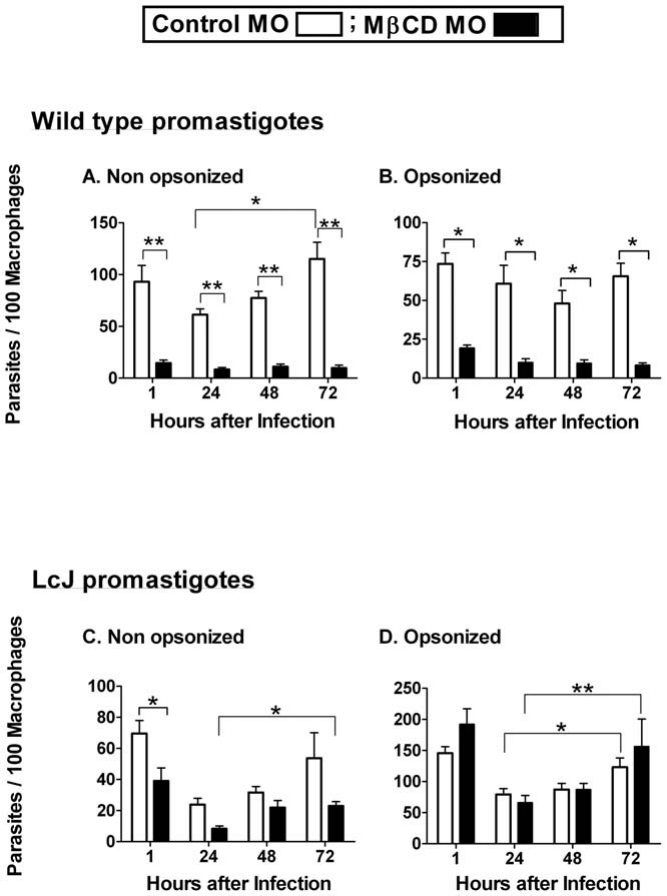
Cholesterol facilitates the entry and intracellular survival of promastigotes. Bone marrow macrophages were either left untreated as a control (open bars) or treated with 10 mM for 1 h (solid bars) to transiently deplete cell membrane cholesterol prior to infection. Separate experiments were performed for wild-type and LcJ promastigotes as well as for non-opsonized and 5% A/J serum opsonized parasites. At the indicated times, cells were fixed and stained with Wright-Giemsa and parasite load was assessed by microscopy. Data indicate parasites per 100 macrophages for wild-type metacyclic (**A** and **B**) and LcJ (**C** and **D**) promastigotes and are the means ± SE of 3 repeats, in triplicates (non-opsonized) or duplicate conditions (serum opsonized). Statistical analysis, (t-test), * P = <0.05, **P = <0.001.

Previous work in our laboratory showed that transient depletion of macrophage membrane cholesterol, under conditions that do not deplete intracellular cholesterol stores, decreases the entry and survival of *L. i. chagasi* stationary phase promastigotes [9]. Stationary cultures are a mixed population containing promastigotes at various levels of virulence [32]. We therefore adapted a gradient separation protocol to isolate the highly infectious metacyclic promastigotes [26], [33] and investigated the role of cholesterol in the entry and survival of metacyclic *L. i. chagasi* promastigotes. Transient depletion of macrophage membrane cholesterol using MβCD reduced the entry of metacyclic *L. i. chagasi* promastigotes and prevented their replication for up to 72 h (Fig. 1A).

CR3 is a macrophage receptor that binds iC3b-coated particles and facilitates the phagocytosis of *Leishmania* spp. promastigotes. CR3 ligation avoids classical macrophage activation and could therefore facilitate intracellular survival [34], [35]. We therefore investigated whether serum opsonization affected the cholesterol-mediated entry of *L. i. chagasi*. To allow C3 deposition without formation of the membrane attack complex (MAC), promastigotes were opsonized with C5-deficient serum from A/J mice. Serum opsonization did not affect the cholesterol-dependent entry or intracellular survival of metacyclic promastigotes (Fig. 1B).

We previously reported that serum opsonization abrogates the effect of cholesterol depletion on the entry of attenuated promastigote into macrophages [9]. LcJ is a stage-cycling *L. i. chagasi*-derived parasite cell line that converts between promastigote and amastigote forms in axenic culture. LcJ promastigotes retain higher virulence than attenuated *L. i. chagasi* parasite lines, although their virulence in mice is lower than that of metacyclic promastigotes [36]. Transient depletion of macrophage cholesterol moderately decreased the entry of non-opsonized LcJ promastigotes, whereas the entry of serum opsonized LcJ promastigotes was not affected by cholesterol depletion (Fig. 1C, 1D). Thus, the entry of LcJ promastigotes was similar to attenuated *L. i. chagasi* (L5), although LcJ parasites retained the ability to replicate in macrophages with or without serum opsonization (Figs. 1C and 1D) [9].

**Figure 2:**
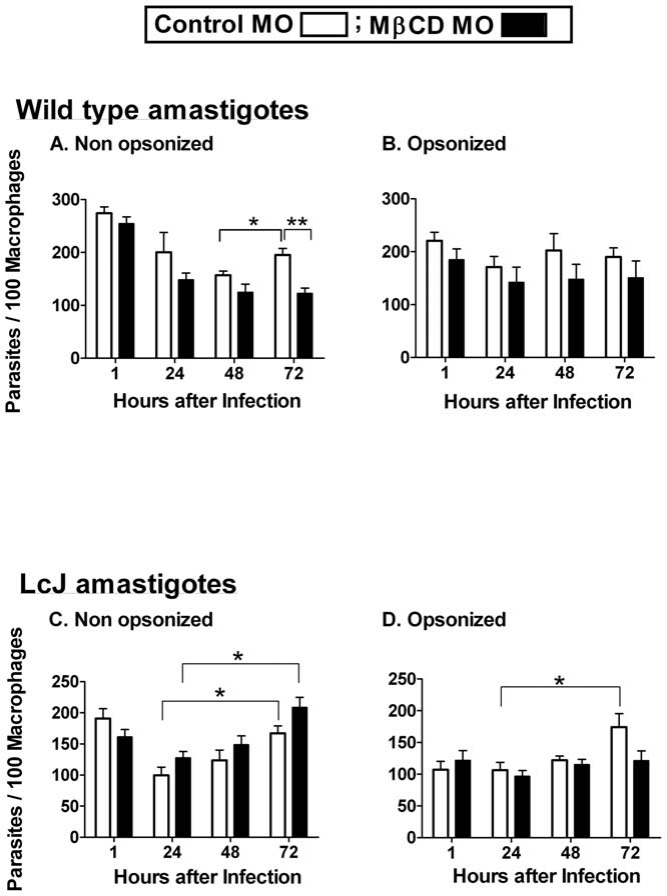
Cholesterol depletion does not affect the entry of amastigotes. Bone marrow macrophages were either left untreated as a control (open bars) or treated with 10 mM MβCD for 1 h (solid bars) prior to infection. Separate experiments were performed for wild-type and LcJ amastigotes as well as for non-opsonized and 5% A/J serum opsonized parasites. Parasite load and intracellular survival was quantified as described. Data indicate parasites per 100 macrophages for wild-type (**A** and **B**) and LcJ (**C** and **D**) amastigotes and are the means ± SE of 3 repeats in triplicates (non-opsonized) or duplicate conditions (serum opsonized). Statistical analysis, (t-test), * P = <0.05, ** P = <0.001.

In contrast to the observations with promastigotes, cholesterol depletion did not affect the phagocytosis or intracellular survival of either hamster-derived or LcJ amastigotes in the initial 48 h of infection (Figs. 2A–2D). Furthermore, in opsonized conditions, the intracellular numbers of either hamster-derived or LcJ amastigotes remained fairly constant for up to 72 h (Figs. 2B and 2D). For non-opsonized parasites, however, cholesterol depletion affected the ability of hamster-derived, but not of LcJ amastigotes to replicate at 72 h (Figs. 2A and 2C). These data suggest that cholesterol can affect wild-type amastigotes by additional downstream effects. Taken together, these cholesterol depletion studies indicate that the highly virulent metacyclic promastigotes are very dependent on macrophage membrane cholesterol for their entry and intracellular survival, but the partially attenuated LcJ promastigotes are less dependent. In contrast, amastigotes do not require intact macrophage cholesterol for efficient phagocytosis or intracellular survival. Nonetheless, by 72 h, the ability of hamster-derived amastigotes to replicate was impaired if entry was initiated in both, the absence of serum and through a cholesterol-disrupted pathway (Fig. 2A).

### Caveolin-1 clusters with promastigotes but not with amastigotes

**Figure 3:**
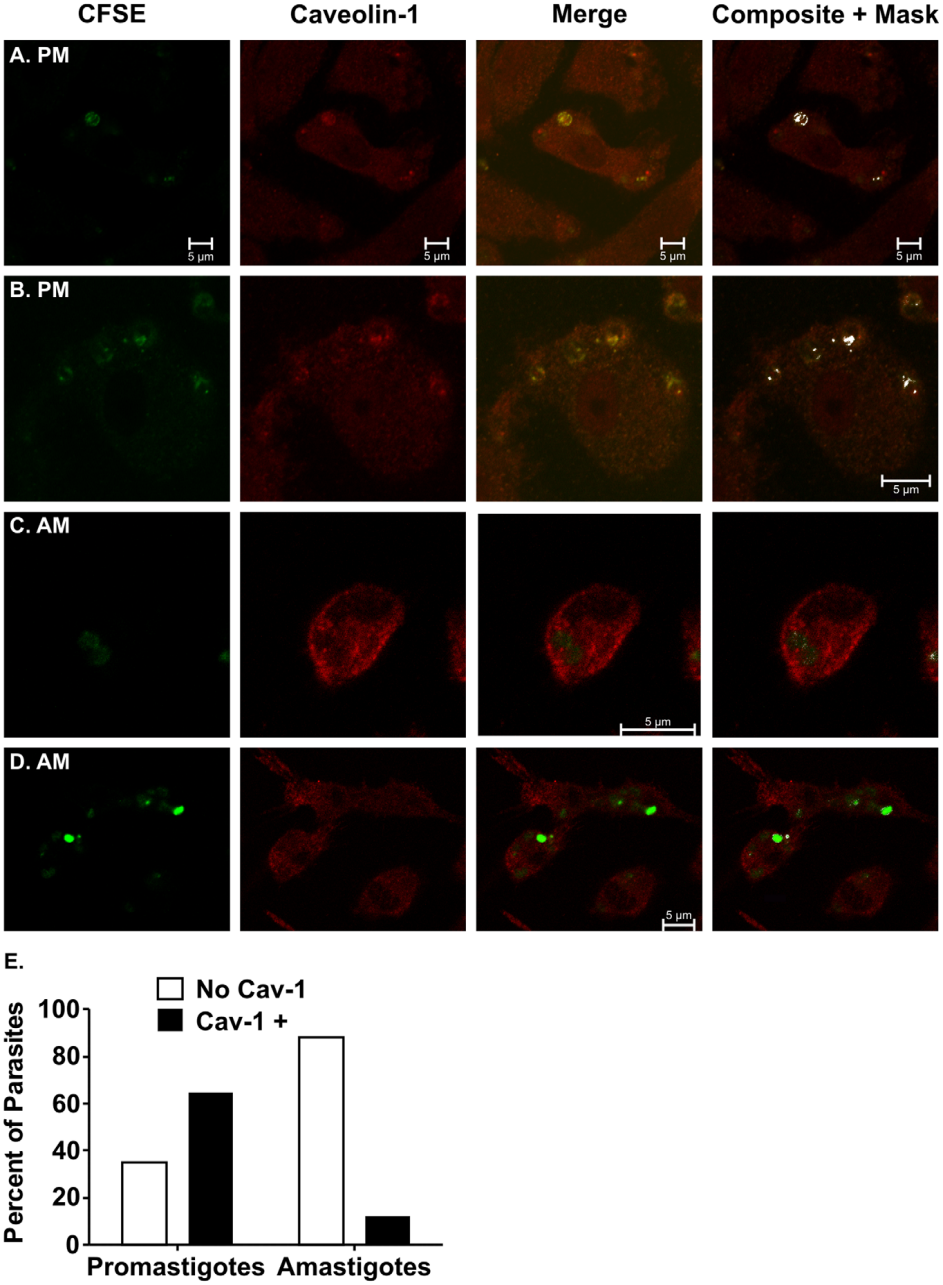
Promastigotes, but not amastigotes, associate with caveolin-1. Macrophages were incubated with either *L. i. chagasi* promastigotes (**A** and **B**) or amastigotes (**C** and **D**). Co-localization of CFSE-labeled parasites (green) with caveolin-1 (red) was assayed by confocal microscopy 1 h after parasite addition. Merged data are shown in the third column, and the fourth column includes Image J “composite and mask” function in which co-localized pixels are shown in white. Images shown are representative of four independent experiments. Scale bar = 10 µm. The “composite and mask” images were used to quantify the proportion of promastigotes or amastigotes that colocalized with caveolin-1 (panel **E**). A total of 329 promastigotes or 200 amastigotes were examined; differences are statistically significant (p<0,001, Chi Square).

Caveolin-1 is the hallmark structural component of caveolae, a subset of lipid rafts. Our group previously reported that macrophage entry through a cholesterol-rich pathway is essential for virulent promastigotes to delay phagosome-lysosome fusion and to replicate intracellularly [9]. Contrary to promastigotes, amastigotes are relatively resistant to the microbicidal properties of lysosomes. Indeed, amastigotes require the phagolysosome environment to survive and replicate [2], [18]. As such, we hypothesized that amastigotes do not benefit from using a cholesterol-rich pathway, like caveolae, to enter and survive in macrophages. To test this premise, we examined the association of promastigotes and amastigotes with caveolin-1. Consistent with our hypothesis, out of 329 promastigotes examined, 65% associated with caveolin-1 clusters (Figs. 3A and 3B) whereas out of 200 amastigotes, 88% failed to do so (Figs. 3C and 3D). The difference in caveolin-1 association between promastigotes and amastigotes is significant (P<0.001, Chi square). These data are consistent with different routes of macrophage entry for promastigotes and amastigotes (Figs. 1 and 2), and suggest that the different entry pathways may uniquely facilitate the intracellular survival of each parasite form.

### Phagosome maturation in promastigote and amastigote-containing PVs

After phagocytosis, phagosomes normally undergo a maturation process that culminates in lysosomal fusion and microbe killing [37]. The rate and extent of phagosome maturation can be altered by ligation of different receptors or by pathogen-initiated disruption mechanisms that favor intracellular survival and disease progression [11]. *Leishmania* spp. amastigotes are relatively resistant to killing by reactive oxygen and reactive nitrogen intermediates, and are able to survive and replicate within the inhospitable environment of the phagolysosome [2]. Because our results suggested that promastigotes and amastigotes differ in their pathway of macrophage entry, we reasoned that this may be coupled to changes in the rate or extent of phagosome maturation.

**Figure 4:**
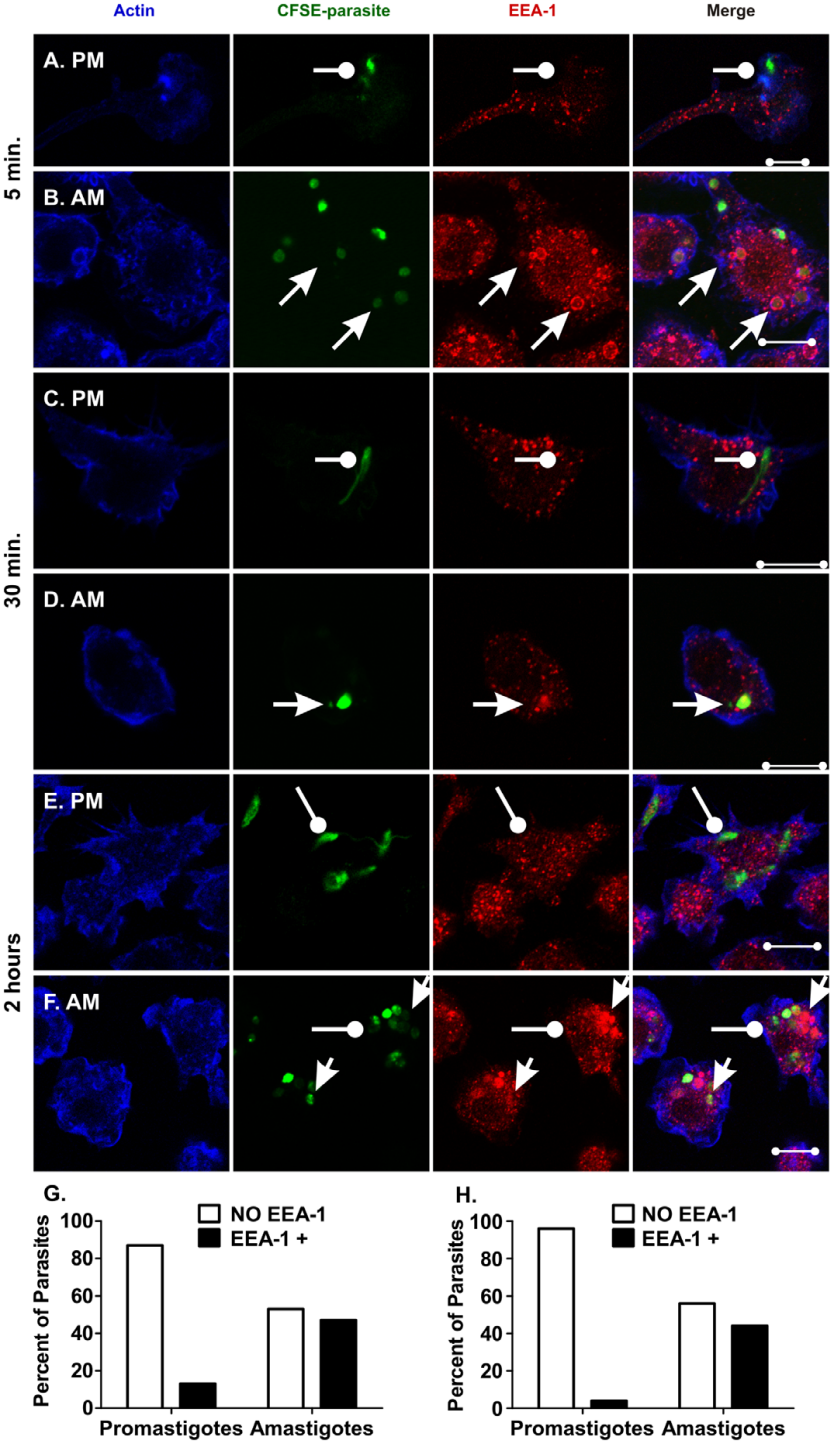
Amastigotes, but not promastigotes retain EEA-1 during infection. Bone marrow macrophages were infected with either LcJ promastigotes (**A**, **C**, and **E**) or LcJ amastigotes (**B**, **D** and **F**) as described. Samples were fixed, stained and examined by confocal microscopy at 5 min. (**A** and **B**), 30 min. (**C** and **D**), or 2 h (**E** and **F**) after infection. Representative parasites associated with EEA-1 are indicated by arrows, and representative parasites not associated with EEA-1 are marked by wands. Panels **A**, **C** and **E** demonstrate a lack of colocalization of promastigotes with EEA-1. Panel **B** shows 6 several “dim” amastigotes that co-localized with EEA-1 and three “bright” amastigotes that did not. The one amastigote visualized in Panel **D** demonstrates co-localization. Panel **F** shows several clusters of amastigotes. Two out of 3 bright amastigotes and 13 out of 15 dim amastigotes colocalize with EEA-1. Blue: actin; Green: CFSE-stained parasites, Red: EEA-1. Yellow indicates EEA-1/parasite co-localization. Scale bar = 10 µm. Panels **G** and **H**: The percentage of promastigotes or amastigotes that associated with EEA-1 was quantified microscopically at either 5 min. (panel **G**: 119 promastigotes, 123 amastigotes) or 2 h (Panel **H**: 68 promastigotes, 159 amastigotes) after addition to macrophages. Statistical analysis showed the EEA-1 differential distribution between parasite stages was significant for each time point (p<0.0001 Chi Square).

The maturation of phagosomes containing *L. i. chagasi* promastigotes or amastigotes was examined in parallel. Using confocal microscopy we assayed the association of parasite-containing phagosomes with markers characteristic of early and late endosomes, i.e., early endosome antigen-1 (EEA-1) or the late endosome/lysosome-associated membrane protein-1 (LAMP-1), respectively [37]. LcJ parasites associated with EEA-1 as early as 5 min. after infection. Examination of 119 promastigotes and 123 amastigotes showed that the number of EEA-1 positive parasites was 13% for promastigotes and 53% for amastigotes (Fig. 4G) (P<0.001, Chi square). Hence promastigotes (Fig. 4A) were less likely than amastigotes (Fig. 4B) to recruit EEA-1 during entry. Similar patterns of EEA-1 association with promastigotes and amastigotes were observed at 15 min. (data not shown) and 30 min. (Figs. 4C and 4D, respectively). At 2 h of infection, we examined 68 promastigotes and 159 amastigotes. By this time, EEA-1-positive early endosomes were dispersed throughout the macrophage cytoplasm and did not associate with most (95%) promastigotes (Fig. 4E). In contrast, 55% of amastigotes were EEA-1 positive (Figs. 4F and 4H). The unusual retention of EEA-1 for up to 2 h is specific to amastigotes, but not to promastigotes (P<0.001, Chi square). Studies of PVs surrounding WT *L. i. chagasi* metacyclic promastigotes yielded similar results (data not shown).

**Figure 5:**
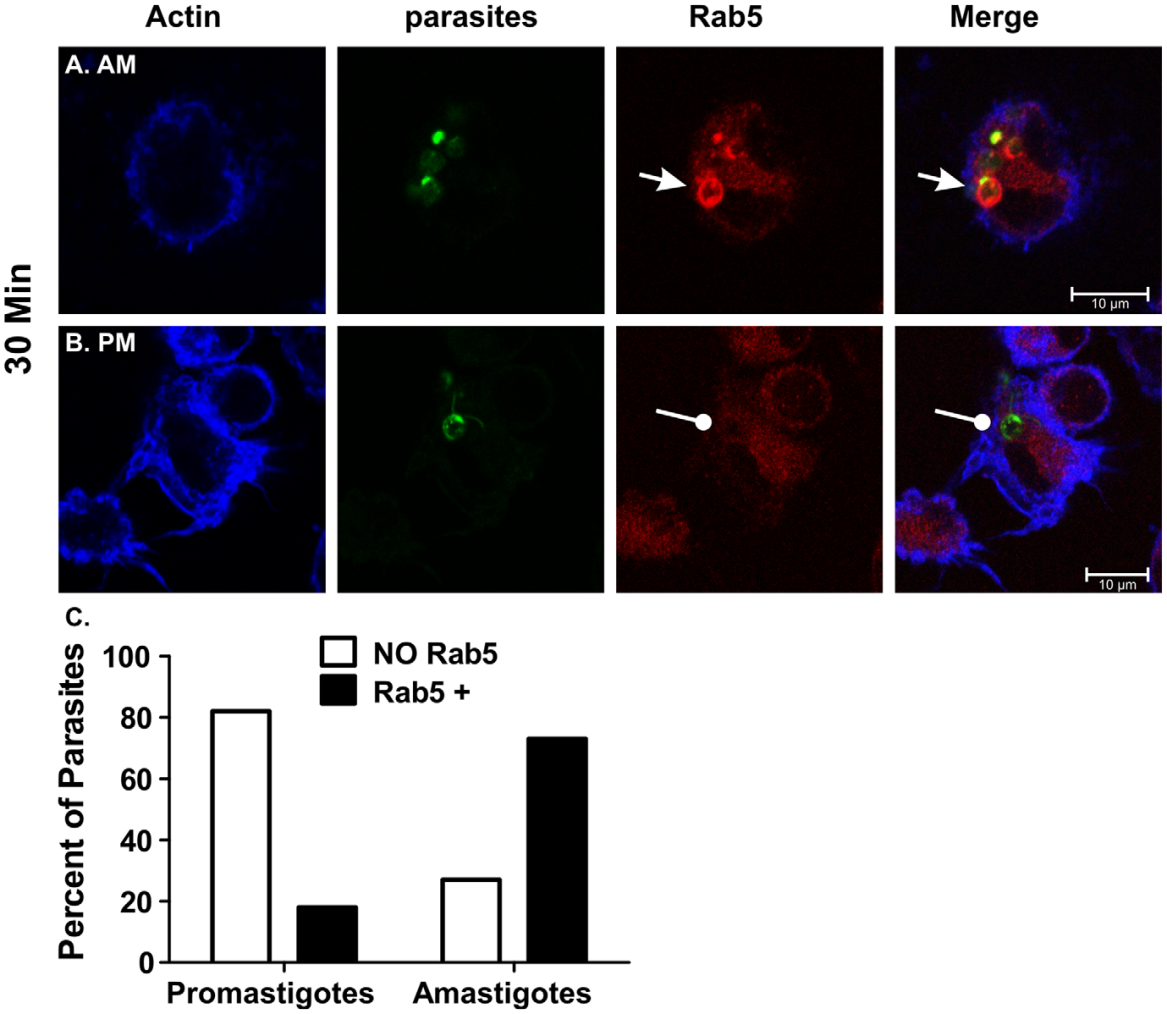
Rab5 accumulates near amastigotes. Bone marrow macrophages were infected with LcJ parasites as described. In the initial 30 min. of infection, Rab5 associates with amastigotes (**A**), but not with promastigotes (**B**). The percentage of promastigotes or amastigotes associating with Rab5 was quantified for 56 promastigotes and 111 amastigotes (**C**). Differences were statistically significant (p<0.005, Chi Square). Blue: actin; Green: CFSE-stained parasites, Red: Rab-5. Yellow indicates Rab-5/parasite co-localization. Scale bar = 10 µm.

An unexpected observation was the accumulation of EEA-1 pools (Fig. 4F). In three replicate experiments, EEA-1 clusters were absent in promastigote-, but present in amastigote-infected cells (Figs. 4E and 4F, respectively). EEA-1 accumulation was detected by 15 min. of amastigote infection and remained for more than 2 h. Many of these clusters were large (average 2–4 µm in diameter, and up to 11 µm^2^) and co-localized with dimly stained amastigotes (Fig. 4F). These results suggested that amastigote infection induced the accumulation and/or retention of compartments resembling early endosomes. To confirm these observations we examined Rab5, another marker of early endosomes. After the initial 30 min. of incubation, 10 (18%) out of 56 promastigotes were positive for Rab5 (Fig. 5B). In contrast, 81 (73%) out of 111 amastigotes were Rab5 positive (Figs. 5A and 5C). The differential accumulation of Rab5 by promastigotes versus amastigotes was significant (P<0.0001; Chi square). By 1 h, 42% of amastigotes still retain Rab5 whereas only 7% of promastigotes did so (data not shown; P<0.005, Chi square). Similar to EEA-1, Rab5 remained associated with amastigote PVs well after the protein was shed by PVs formed around promastigotes. Likewise, most Rab-5-positive vacuoles contained amastigotes with only dim CFSE stain (Fig. 5A).

The presence of dimly stained amastigotes agrees with our initial studies that showed two types of intracellular amastigotes: one brightly stained with CFSE (green) and one with faded CFSE staining. In contrast, extracellular amastigotes were bright green (data not shown), suggesting that the CFSE fading is an infection-related phenomenon. Our CFSE stained parasites were visualized by exciting the fluorescent probe at 488 nm. Studies in bacteria demonstrated that at an excitation wavelength of 490 nm, CFSE is pH sensitive [38]. Thus, it is most likely that the dim parasites represent those amastigotes whose compartments are maturing rapidly. In addition, the pH sensitivity of CFSE might explain why the observation of dim parasites was a phenomenon more readily observed with amastigotes than with promastigotes, whose phagosomes matured slowly. To circumvent this problem, we stained parasites with the nuclear stain TOPRO-3. Co-staining with CFSE and TOPRO-3 showed nuclei and kinetoplasts in bright and dim green as well as amastigotes with no CFSE staining (data not shown). Moreover, the number of bright CFSE-stained amastigotes decreased as the infection progressed. Because microscopic counts revealed amastigotes replicated in these cultured cells, we surmise that at least, most of these parasites remained viable but lost cytoplasmic CFSE stain as part of their intracellular phase.

**Figure 6:**
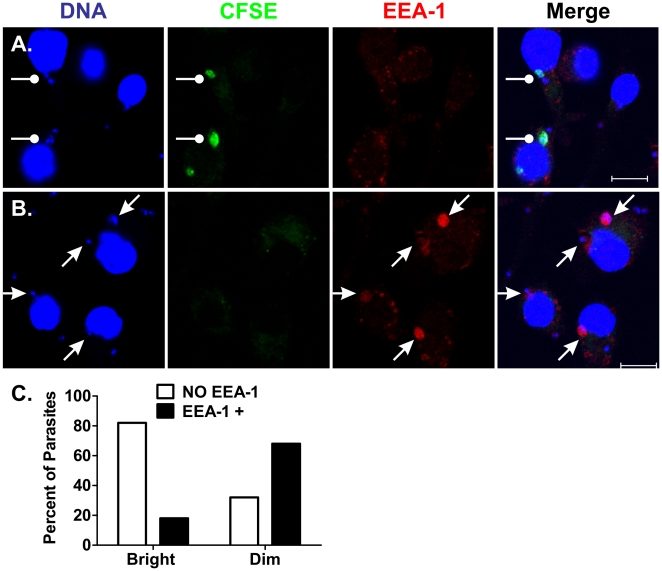
Differential EEA-1 accumulation between bright and dim amastigotes. Macrophages were incubated with CFSE-stained amastigotes (green) for 30 minutes at 37°C, 5% CO_2_. Cells were then stained with TO-PRO-3 (blue) which stained both macrophage and amastigote nuclei, and amastigote kinetoplasts, and stained for EEA-1 (Red). Panels **A** and **B** show examples of bright green (CFSE) amastigotes, most of which lacked EEA-1 co-localization (**A**) and “dim” (faintly or not green) amastigotes, most of which accumulated EEA-1 (**B**). The proportion of bright or “dim” amastigotes associated with EEA-1 was quantified in 227 amastigotes (**C**). Differences were statistically significant (p<0.0001, Chi square).

The brightness of CFSE staining was quite variable among intracellular amastigotes. Hypothesizing that we might not detect all “dim” amastigotes, we incubated macrophages with CFSE-stained parasites and subsequently stained with the DNA stain TOPRO-3, to mark the amastigote nuclei and kinetoplasts. Indeed, 30 min. after amastigote addition, only 10% of the 227 amastigotes examined retained their bright CFSE staining whereas the remaining amastigotes were dim or “unstained” and only visible by DNA staining (Figs. 6A and 6B). Among the amastigotes that retained their bright CFSE stain, 80% were EEA-1 negative and only 20% were EEA-1 positive (Figs. 6A and 6C). In contrast, 90% of amastigotes became dim or “unstained” with CFSE. Among these, 29% were EEA-1 negative and 61% EEA-1 positive at (Figs. 6B and 6C). The differential association of EEA-1 with bright versus dim/unstained amastigotes was statistically significant (P<0.0001, Chi square).

**Figure 7:**
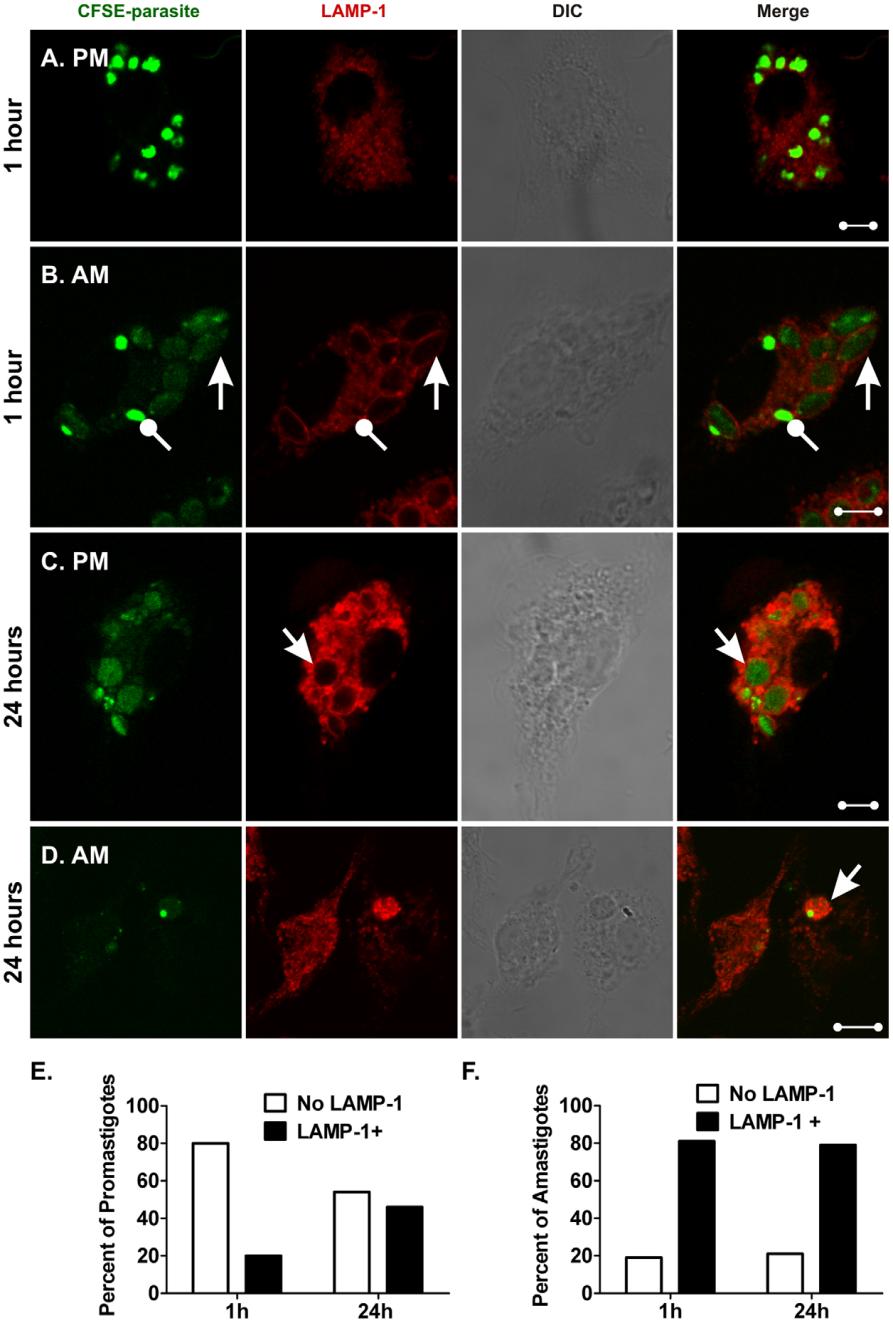
Parasitophorous vacuoles containing promastigotes and amastigotes recruit LAMP-1 with different kinetics. Macrophages were infected with *L. i. chagasi* metacyclic promastigotes (**A** and **C**) or hamster-derived amastigotes (**B** and **D**). Samples were fixed, stained and examined by confocal microscopy after 1 h (**A** and **B**) or 24 h (**C** and **D**). Images show CFSE-stained parasites in green and LAMP-1 in red. Bright green, LAMP-1-negative amastigotes are marked with a wand, and dim, LAMP-1+ amastigotes are indicated by arrows (**B**). DIC: differential interference contrast. Scale bar = 5 µm. Images are representative of multiple cells in five replicate experiments. Panels **E** and **F** demonstrate graphically the proportions of metacyclic promastigotes (panel **E**) or hamster-derived amastigotes (panel **F**) residing in LAMP-1+ or LAMP-1-negative compartments at 1 h or 24 h after parasite inoculation. Numbers were derived from examining 194 promastigotes and 340 amastigotes. Differential distribution of LAMP-1 in promastigote PVs at the two time points (panel **E**) was statistically significant (P<0.001, Chi square). In contrast, there was no difference between the distribution of LAMP-1 in amastigote PVs at the two time points (panel **F**), (p = NS, Chi square).

Further maturation of PVs was assessed using antibodies to LAMP-1, a marker of late endosomes and lysosomes [37]. Parallel samples stained 1 h after the addition of either metacyclic promastigotes or hamster-derived amastigotes were distinct. Metacyclic promastigotes were primarily in LAMP-1 negative compartments (Fig. 7A). In contrast, amastigote-infected cells contained two types of PVs. LAMP-1-negative compartments contained brightly stained CFSE-labeled parasites, whereas LAMP-1+ compartments contained dimly stained CFSE-labeled parasites (Fig. 7B). These results suggest that amastigotes lose CFSE stain when in the maturing endosomal compartment. Twenty-four hours after infection was initiated, many promastigote-induced PVs were LAMP-1+, indicating fusion with late endosomes/lysosomes (Fig. 7C). As predicted by literature reports, after 24 h, PVs initiated by amastigote infection were mostly LAMP-1+ (Fig. 7D) [12]. More than six replicate studies of both WT and LcJ parasites demonstrated similar kinetics of LAMP-1 recruitment to the PV, i.e., fast in amastigote- and delayed recruitment in promastigote-induced PVs.

We examined 194 metacyclic promastigote- and 340 amastigote-containing PVs and quantified LAMP-1 recruitment at 1 h and 24 h after infection. At 1 h post-infection, 80% of the promastigotes resided in LAMP-1-negative PVs whereas 20% were LAMP-1+. By 24 h, 46% of promastigote PVs were LAMP-1+ (Fig. 7E), a statistically significant increase (P<0.001, Chi square). By 1 h of infection, the distribution of amastigotes in LAMP-1-negative and LAMP-1+ compartments was 19% and 81%, respectively. Twenty-four hours later, there was no significant change, at 21% and 79%, respectively (Fig. 7F). Similar results were obtained with LcJ parasites (data not shown). This comparison between metacyclic promastigotes and infection-derived amastigotes support a model in which the kinetics of phagosome maturation depends upon the parasite stage initiating the infection.

### Disruption of macrophage cholesterol microdomains accelerates the recruitment of LAMP-1 to promastigote, but not to amastigote-containing phagosomes

**Figure 8:**
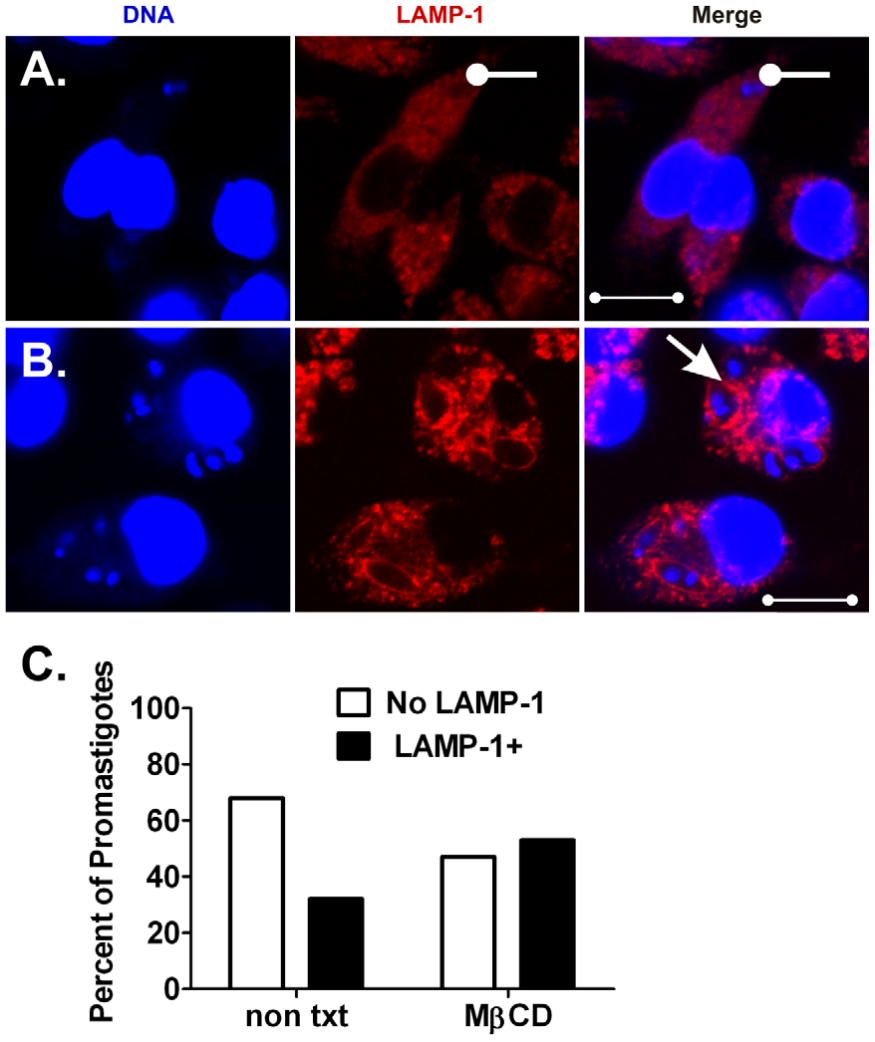
Transient disruption of macrophage lipid rafts accelerates LAMP-1 fusion with promastigote compartments. Macrophages were either left untreated as a control (**A**) or treated with 10 mM MβCD for 1 h (**B**) prior to infection with LcJ promastigotes. After 1 h, samples were fixed and stained for confocal microscopy. Tight, LAMP-1 negative or spacious, LAMP-1+ compartments are marked by wands or arrows, respectively. Data shown are representative of three independent experiments. Blue: TOPRO-3 stained DNA, Red: LAMP-1. Scale bar = 10 µm. (**C**) Amongst 487 intracellular promastigotes examined, the proportion residing in LAMP-1 negative versus LAMP-1+ compartments was significant (P = <0.001, Chi square).

**Figure 9:**
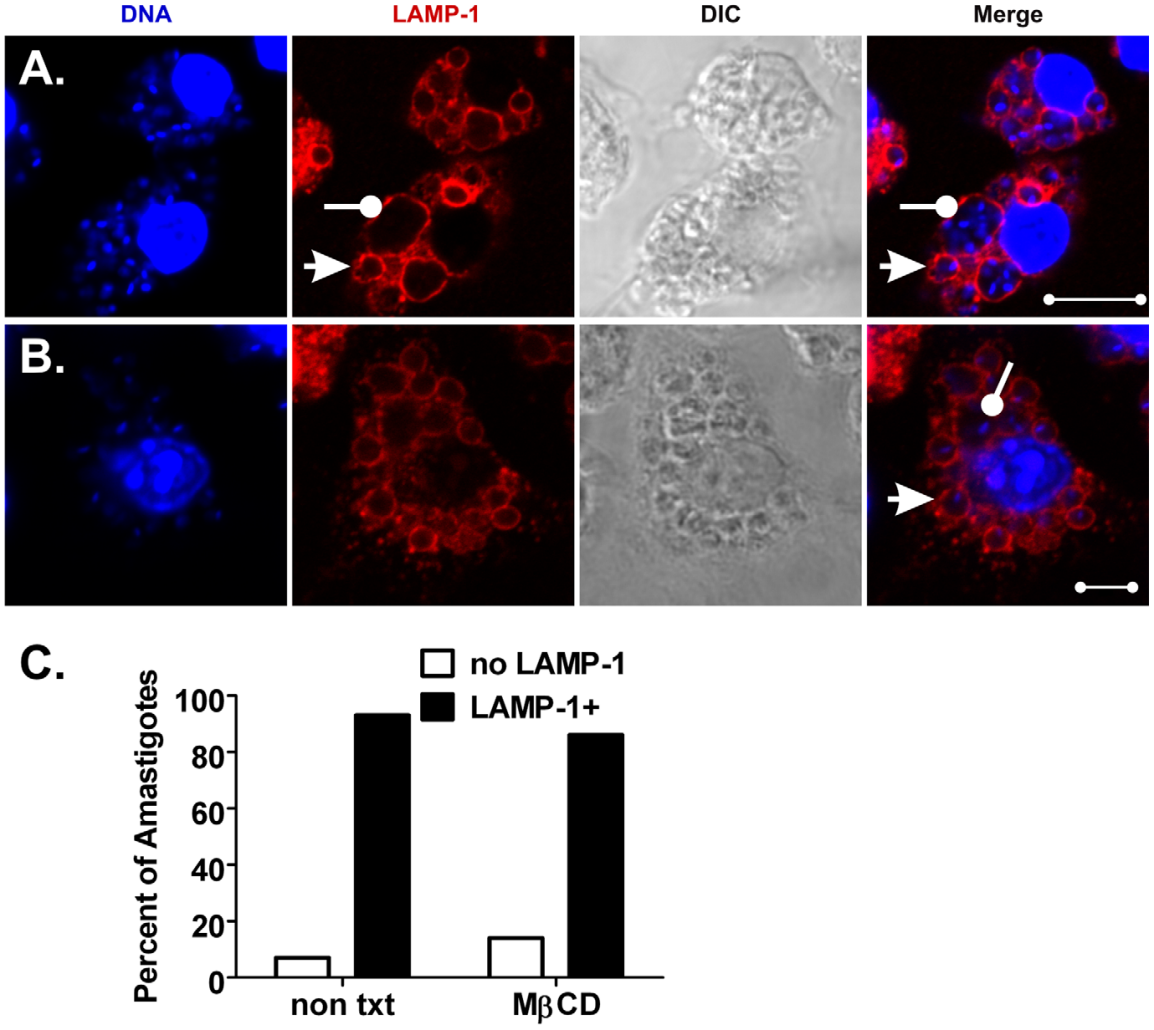
Fusion of LAMP-1 with amastigotes compartments is independent of lipid raft integrity. Macrophages were either left untreated as a control (**A**) or treated with 10 mM MβCD for 1 h (**B**) and then infected with LcJ amastigotes for 1 h. Fixed cells were processed for confocal microscopy. LAMP-1+ compartments containing single and multiple parasites are indicated by arrows and wands, respectively. Blue: DNA, Red: LAMP-1. Scale bars, (A) = 5 µm; (B) = 10 µm. Panel (**C**) show a graphical representation of the numbers of amastigotes residing in LAMP-1 negative or LAMP-1+ compartments after examination of 461 or 530 amastigotes from control or treated macrophages, respectively. Differences were not statistically significant between the time points (p = NS, Chi square). Data were derived from three separate experiments.

Previously, we showed that entry of virulent *L. i. chagasi* promastigotes into macrophages through lipid rafts facilitates parasite survival at least in part by delaying PV-lysosome fusion [9]. As demonstrated in Figures 8 and 9, we compared the effects of lipid raft disruption on the fusion of lysosomes with promastigote or amastigote-containing PVs. Untreated (control) or MβCD-treated (cholesterol-depleted) macrophages were incubated with either LcJ promastigotes or amastigotes and assayed for phagosome maturation 1 h after infection. Analysis of random fields of cells confirmed that promastigote-initiated PVs in control macrophages were predominantly tight (close-fitting), LAMP-1-negative compartments (Fig. 8A). In contrast, promastigote compartments in MβCD-treated macrophages were enlarged (spacious) and rapidly acquired LAMP-1 as we previously reported (Fig. 8B), [9]. LAMP-1 fusion was evaluated in 487 promastigote phagosomes; 307 from non-treated and 180 from MβCD-treated macrophages. Transient disruption of cholesterol from membrane microdomains increased the accumulation of LAMP-1 on promastigote-induced PVs from 22% to 53% (Fig. 8C, P<0.001, Chi square).

In contrast to promastigotes, amastigote-containing PVs fused with LAMP-1 within 1 h, even in the absence of MβCD treatment (Figs. 9A and 9B). Quantitative analysis of 461 amastigotes in control and 530 amastigotes in MβCD-treated macrophages showed similar rates of parasite recruitment into LAMP-1+ compartments, i.e., 93% and 86%, respectively (Fig. 9C). Most LAMP-1 positive compartments were oval and about 2 µm in diameter, conforming to the amastigote shape [39]. However there were a few large communal compartments (up to 10 µm in diameter and 50 µm^2^) that appeared to contain more than one parasite, observed only in macrophages incubated with amastigotes but not with promastigotes (compare Figs. 8 and 9). Whether these communal vacuoles resulted from a failure to form new PVs upon amastigote replication, fusion of several PVs or from phagocytosis of amastigote clumps, is not known.

## Discussion

Phagocytosis of *Leishmania* spp. by macrophages is initiated by promastigote ligation of several receptors including the fibronectin receptor, the mannose receptor and CR3. The latter binds iC3b, the inactive form of C3b. Ligation of at least some types of CR3-coated particles leads to down-modulation of macrophage anti-microbial responses [3], [4], [35], [40], and could thereby facilitate parasite survival. During the initial 48–72 h of infection, promastigotes convert into the replicative amastigote form. It has been claimed that amastigotes are released by rupture of heavily laden macrophages and are subsequently internalized by nearby non-infected macrophages, hence disseminating the infection [41], [42]. Although not well characterized, amastigote phagocytosis has been reported to occur through macrophage receptors for Fc-γ and phosphatidylserine [5], [6], [43], [44]. In general, ligation of host cell receptors by microbial ligands can result in distinct mechanisms of phagocytosis and signaling [21], [22], [45]. We therefore hypothesized that the mechanism of uptake of each of the two stages of *Leishmania*, and the resulting macrophage responses may have important implications for the outcome of infection.

We previously showed that macrophage microdomains enriched in cholesterol and caveolin-1 facilitate the entry and survival of stationary phase *L. i. chagasi* promastigotes [9]. Using purified metacyclic promastigotes [26] and *in vitro* generated as well as animal-derived amastigotes, we re-examined the requirement for cholesterol-enriched microdomains in the entry and fate of both parasite life stages in murine macrophages. Because of the importance of CR3 for promastigote phagocytosis, we also studied the role of serum opsonization in cholesterol-mediated phagocytosis of each of the parasite stages.

Both entry and intracellular survival of metacyclic *L. i. chagasi* promastigotes were sensitive to cholesterol depletion, whether the parasites were opsonized or not. For the partially attenuated LcJ parasites, cholesterol depletion only had a moderate effect on the entry of non-opsonized promastigotes. In contrast, serum opsonized LcJ promastigotes were resilient to cholesterol depletion, similar to our results with attenuated promastigotes [9]. These observations led us to hypothesize that the usual entry path for metacyclic promastigotes leads to efficient uptake, delayed PV fusion with lysosomes, and consequent intracellular survival. Presumably phagocytosis involves CR3 through either local or exogenous opsonization with iC3b. Metacyclic promastigotes entering macrophages with disrupted lipid microdomains may be directed toward an intracellular pathway that leads to accelerated parasite death. Since LcJ promastigote cultures contain a mixture of metacyclic and non-virulent promastigotes and their virulence is intermediate between wild-type and attenuated lines [25], LcJ promastigotes would not uniformly enter through the cholesterol raft-dependent CR3 pathway thereby explaining their diminished sensitivity to cholesterol depletion.

The promastigote-specific LPG favors intracellular survival in part by delaying fusion with lysosomes [11], [14]. Amastigotes do not display LPG. Furthermore, amastigotes replicate in the phagolysosome environment and likely resist their microbicidal activity through different mechanisms [12], [18], [46]. Transient cholesterol depletion from the macrophage membrane did not affect the entry of hamster-derived or LcJ amastigotes whether they were opsonized or not. These results agree with earlier studies showing that amastigotes are not efficiently opsonized with complement proteins and do not efficiently bind to CR3 [3]. Overall, LcJ amastigotes or serum opsonized hamster-derived amastigotes were not adversely affected by cholesterol depletion. Nonetheless, by 72 h, replication of hamster-derived amastigotes was impaired in macrophages that were pre-treated with MβCD, but only under non-opsonized conditions. We previously showed that lipid rafts at the cell surface are restored 4 h after MβCD treatment and does not affect the viability of the macrophages [9]. Therefore, these unexpected results suggest two possibilities. First, depletion of cholesterol-containing lipid rafts could facilitate phagocytosis of hamster-derived amastigotes through a pathway that allows slow killing. Second, macrophage cholesterol might have additional effects on lysosomes other than modulating their fusion with PVs, such as altering their composition or function. In this way, transient depletion of cholesterol before infection might affect the ability of parasites to acquire nutrients and replicate in the phagolysosome.

Our studies showed that for promastigotes, the kinetics of acquiring early lysosome markers was similar to the kinetics of promastigote to amastigote conversion [42]. Thus, promastigote PVs delayed acquisition of LAMP-1 for at least 24 h after infection, whereas most amastigotes resided in PVs that acquired LAMP-1 quickly after infection. Further investigations of intracellular trafficking of promastigotes versus amastigotes revealed that the latter do not co-localize with caveolin-1, suggesting that amastigotes do not enter macrophages through caveolae. This was true for both hamster-derived and axenic LcJ amastigotes, and therefore was not attributable to an effect of residual hamster membranes or IgG from the host. Consistent with this, the entry of promastigotes was sensitive to the disruption of lipid raft microdomains including caveolae, but uptake of amastigotes was not.

Stage-specific differences in the maturation of promastigote and amastigote phagosomes were revealed using antibodies specific for markers of early endosomes, i.e. the small GTPase Rab-5 and its effector, EEA-1. Vacuoles containing each parasite stage acquired these markers at the earliest time point examined (5 min.), indicating they had entered the endosomal pathway. Consistent with the work of Courret *et al.* [17] in *L. amazonensis* infection, the association of promastigote-induced PVs with EEA-1 was modest and transient. In contrast, most amastigotes associated with EEA-1 for at least 2 h after infection. The dichotomous association of caveolin-1 and EEA-1 with promastigotes versus amastigotes is consistent with studies showing that particle uptake through caveolae results in endosomes positive for caveolin-1 and negative for EEA-1. In contrast, entry through clathrin-coated pits, a non-lipid raft pathway, results in endosomes negative for caveolin-1 and positive for EEA-1 [47]. It will be of interest to determine whether or when amastigote-induced PVs shed EEA-1, and whether PVs induced by promastigotes re-acquire the EEA-1 marker after intracellular conversion into amastigotes. Studies such as these may indicate whether EEA-1 is important for the long-term intracellular survival of amastigotes.

Stage specific differences were also observed in the association of parasites with Rab5, another marker of early endosomes. By 30 min. of infection, amastigotes, but not promastigotes, retained Rab5 staining. These results agree with studies in *L. mexicana* infection, in which amastigotes retain Rab5 longer than promastigotes, i.e., about one and two minutes, respectively [48]. The ability of parasites to hold on longer to Rab5 is LPG mediated, as LPG KO promastigotes retained Rab5 similar to amastigotes. Reminiscent of our observation of large Rab5 clusters in amastigote-infected cells, Lippuner *et al.* [48] reported fusion of Rab5+ parasite compartments. Interestingly, in these fused compartments, Rab5 is retained for at least 30 min., agreeing with our observations.

The significance of Rab5 retention in amastigote compartments might be explained in light of its cellular function. Rab proteins are small GTPases that confer specificity to membrane fusion events in the endosomal pathway. During maturation of phagosomes containing model particles a “kiss and run” fusion mechanism mediates interactions of these compartments with organelles of the endosomal pathway. Rab5 is required for phagosome fusion with early endosomes [49]. Overexpression of Rab5a in J774 macrophages accelerates maturation of *L. monocytogenes* phagosomes without affecting phagocytosis, whereas depletion of Rab5a has the opposite effect [50]. In seeming contrast, expression of the constitutively active Rab5c in HeLa cells prolongs retention of Rab5 on live, but not heat-killed *M. tuberculosis*-containing compartments and correlates with impaired phagosome maturation [51].

Further evidence for the role of Rab5 in pathogen survival comes from *L. donovani* studies in cells transfected with a constitutively active form of Rab5 [52]. Upon infection with *L. donovani* promastigotes, transfected, but not control RAW264.7 cells, undergo phagosome fusion resulting in giant phagosomes with low anti-microbial activity despite acquiring early lysosome markers. Furthermore, these giant phagosomes can contain several parasites, similar to our observations with LcJ and hamster-derived amastigotes. Whether amastigotes retain Rab5 (or specific Rab5 isoforms) as a mechanism to alter phagosome maturation and facilitate their survival is unclear at this time.

Macrophage infections initiated with amastigotes displayed two types of PVs: (1) tight, conforming to the parasite shape, LAMP-1 negative compartments harboring parasites that retained their bright CFSE staining, and (2) LAMP-1 positive compartments containing parasites that had lost most of their CFSE staining. These results are reminiscent of studies showing that neutrophils infected with *L. donovani* promastigotes contain two types of PVs, i.e., tight and spacious [53]. In neutrophils, parasites that associated with ER markers were directed to tight, non-degradative compartments, whereas spacious compartments fused with lysosomes and granules leading to parasite degradation.

More recently, a new type of non-degradative compartment has been described in neutrophils infected with *L. major* or *L. donovani* promastigotes [54]. Parasite compartments readily fused with lysosome-like azurophilic granules while avoiding fusion with specific and tertiary granules containing the NADPH oxidase complex. Our studies in macrophages suggest that amastigote localization in LAMP-1+ compartments does not affect viability, as parasites replicate for up to 72 h. The differences between these studies could reflect (1) the differential microbicidal capacities of the neutrophil versus the macrophage (2) the increased resilience of amastigotes, compared to promastigotes, to the toxic effects of reactive oxygen and nitrogen radicals [2], [55], [56]. Our results are also consistent with the ability of amastigotes, but not promastigotes, to tolerate rapid phagosome-lysosome fusion.

The two life stages of *L. i. chagasi* differ considerably in their utilization of macrophage membrane domains for entry and subsequent trafficking through the endosomal pathway.

The current study suggests that macrophage phagocytosis of amastigotes does not require the receptors localized in cholesterol-rich lipid raft domains that are so effective in mediating promastigote uptake. Once inside the macrophage, parasite survival is facilitated by differential kinetics of maturation of the PVs surrounding promastigotes and amastigotes during the first hours to days of infection. In contrast to studies showing that phagosomes containing promastigotes and amastigotes of *L. amazonensis* mature at a similar rate, our work with *L. i. chagasi* and that of others with *L. major* and *L. donovani*, demonstrate that maturation of promastigote PVs is delayed [9], [11], [14]. We report here that maturation of amastigote-induced PVs differ from that of promastigote PVs by faster fusion with LAMP-1 and prolonged retention of some early endosome markers. At present, we can only speculate on the role and potential significance of prolonged Rab5 and EEA-1 association with amastigote-containing PVs. Further investigations are needed to identify the specific amastigote ligands and macrophage receptors responsible for the prolonged retention of early endosome markers on PVs as well as their potential effects on parasite fate and virulence.
